# COVID-19 increases the risk for the onset of atrial fibrillation in hospitalized patients

**DOI:** 10.1038/s41598-022-16113-6

**Published:** 2022-07-14

**Authors:** Jakob Wollborn, Sergey Karamnov, Kara G. Fields, Tiffany Yeh, Jochen D. Muehlschlegel

**Affiliations:** Department of Anesthesiology, Perioperative and Pain Medicine, Brigham and Women’s Hospital, Harvard Medical School, 75 Francis St, Boston, MA 02115 USA

**Keywords:** Atrial fibrillation, Viral infection

## Abstract

COVID-19 is associated with significant extrapulmonary symptoms. Myocardial involvement has been described for infections with SARS-CoV-2 which may lead to an increase in morbidity and mortality. The objective of our study was to investigate the association of COVID-19 and atrial fibrillation (AF) or atrial flutter (AFl) in hospitalized patients. This retrospective study used electronic medical records to detect patients with COVID-19 and their comorbidities within the Mass General Brigham hospital system. All patients ≥ 18 years who were hospitalized and received a PCR test for SARS-CoV-2 were screened for inclusion as well as patients from a pre-pandemic cohort. We matched on common risk factors for AF and then used multivariable logistic regression to estimate the odds for AF or AFl. Of 78,725 patients eligible for analysis, 11,004 COVID-19 negative patients were matched to 3,090 COVID-19 positive patients and 5005 pre-pandemic patients were matched to 2283 COVID-19 positive patients. After adjusting for demographics and comorbidities, COVID-19 positive patients had 1.19 times the odds (95% CI 1.00, 1.41) of developing AF compared to COVID-19 negative patients and 1.57 times the odds (95% CI 1.23, 2.00) of developing AF compared to pre-pandemic patients. Our study demonstrated an increased risk for AF, directing the attention for improved screening and treatment regimens for the sequelae of COVID-19. While COVID-19 continues to affect many people around the world, AF may be a significant cause for morbidity and mortality. Adequate detection and treatment of AF is essential to reduce the burden of disease.

## Introduction

Patients suffering from COVID-19 predominately exhibit pulmonary symptoms. Additional effects of the virus on other organ systems have been reported. Specifically, evidence point towards myocardial involvement^1^. Molecularly, direct cellular damage is caused to sarcomere structures^2^. Stressing its clinical impact, cardiac MRI showed signs of myocarditis even in previously asymptomatic athletes who were infected^3^.

In general, cardiac disease is often accompanied by heart rhythm disorders, which increase the likelihood for further complications. Arrhythmias have previously been reported in infections with COVID-19^4^. The most common cardiac arrhythmia is atrial fibrillation (AF) with myocardial inflammation increasing the risk for the development of AF^5^. In AF the risk for complications is elevated—the odds for an ischemic stroke is increased by fivefold^6^, with further thromboembolic events possible (e.g. mesenteric ischemia, myocardial infarction).

The link of COVID-19 and AF needs to be verified. In this retrospective cohort study we hypothesized that COVID-19 positive patients hospitalized between 3/1/2020 and 2/28/2021 would have a greater odds of in-hospital AF compared to COVID-19 negative patients hospitalized during the same time period as well as pre-pandemic historical controls.

## Results

**Figure 1:**
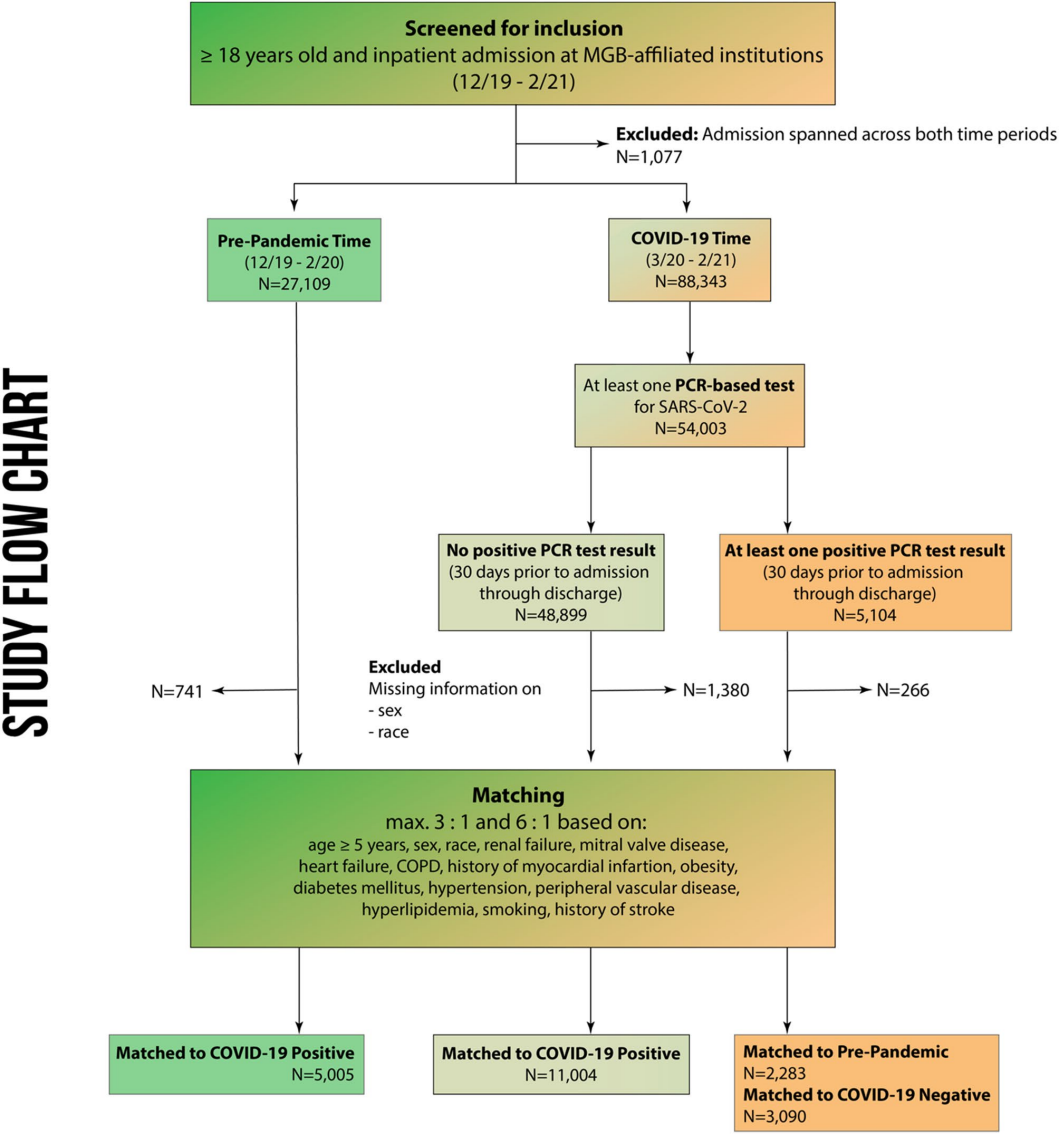
Patient selection and study flow chart.

We identified 116,529 patients who met the study’s inclusion criteria. After exclusions (admissions that spanned across the pre-pandemic and pandemic periods, no valid SARS-CoV-2 PCR test result in the pandemic era, or missing patient information on sex or race), we matched 3090 patients with a positive test for COVID-19 to 11,004 patients with a negative test for COVID-19, and to 5005 pre-pandemic patients in a sensitivity analysis (Fig. 1).

### Demographics

Our demographics of unmatched patients showed 27,447 (out of 47,519; 57.8%) females in the COVID-19 negative cohort compared to 2281 females (out of 4838; 47.1%) in the COVID-19 positive group (*P* < 0.001; standardized difference − 0.2137; see Table 1). In the pre-pandemic cohort, 14,887 patients (out of 26,368; 56.5%) were female (*P* < 0.001; standardized difference − 0.187 vs. COVID-19 positive). Patients who suffered from COVID-19 were older compared to COVID-19 negative patients (mean ± standard deviation [SD] 63 ± 19 vs. 58 ± 21; standardized difference 0.182; *P* < 0.001) and pre-pandemic patients (59 ± 20; standardized difference 0.257; *P* < 0.001).

**Table 1:** Unmatched patient characteristics.

	COVID-19		*P*-value [COVID-positive vs. negative]	Pre-pandemic (n = 26,368) (Count. %)	*P*-value [COVID-positive vs. pre-pandemic]
	Negative (n = 47,519) (Count. %)	Positive (n = 4838) (Count. %)			
Female	27,447 (57.8%)	2281 (47.1%)	**< 0.001**	14,887 (56.5%)	**< 0.001**
Age [years, mean ± SD]	58 ± 21	63 ± 19	**< 0.001**	59 ± 20	**< 0.001**
Asian	2176 (4.6%)	223 (4.6%)	**< 0.001**	1098 (4.2%)	**< 0.001**
Black	4308 (9.1%)	776 (16%)		2244 (8.5%)	
Hispanic	740 (1.6%)	243 (5%)		406 (1.5%)	
White	37,147 (78.2%)	2737 (56.6%)		21,142 (80.2%)	
Other	3148 (6.6%)	859 (17.8%)		1478 (5.6%)	
Chronic renal failure	9200 (19.4%)	1130 (23.4%)	**< 0.001**	6435 (24.4%)	0.118
Mitral valve disease	6708 (14.1%)	473 (9.8%)	**< 0.001**	4464 (16.9%)	**< 0.001**
Congestive heart failure	5860 (12.3%)	570 (11.8%)	0.267	4094 (15.5%)	**< 0.001**
COPD	5860 (12.3%)	592 (12.2%)	0.847	3935 (14.9%)	**< 0.001**
History of myocardial infarction	2004 (4.2%)	201 (4.2%)	0.836	1614 (6.1%)	**< 0.001**
Obesity	13,130 (27.6%)	1617 (33.4%)	**< 0.001**	8359 (31.7%)	**0.018**
Diabetes mellitus	10,060 (21.2%)	1704 (35.2%)	**< 0.001**	6191 (23.5%)	**< 0.001**
Hypertension	25,525 (53.7%)	2971 (61.4%)	**< 0.001**	15,317 (58.1%)	**< 0.001**
Peripheral vascular disease	7025 (14.8%)	634 (13.1%)	**0.002**	4439 (16.8%)	**< 0.001**
Hyperlipidemia	19,208 (40.4%)	2260 (46.7%)	**< 0.001**	11,852 (44.9%)	**0.023**
Smoking	11,628 (24.5%)	811 (16.8%)	**< 0.001**	8142 (30.9%)	**< 0.001**
History of stroke	33 (0.1%)	5 (0.1%)	0.394	25 (0.1%)	0.802
History of AF/AFl	6261 (13.2%)	574 (11.9%)	**0.01**	4506 (17.1%)	**< 0.001**

*AF* atrial fibrillation, *AFl* atrial flutter, *COPD* Chronic obstructive pulmonary disease.

Significant values are in [bold].

Both groups of comparisons showed differences in the proportion of racial groups (*P* < 0.001; standardized differences 0.492 and 0.533 respectively). The COVID-19 positive patients had a higher proportion of Blacks (776 out of 4838; 16%), Hispanics (243 out of 4838; 5%) and others (859 out of 4838; 17.8%) with a smaller proportion of Whites (2737 out of 4838; 56.6%), while both COVID-19 negative and pre-pandemic patients showed a higher proportion of Whites (37,147 out of 47,519 [78.2%] and 21,142 out of 26,368 [80.2%] respectively).

Among the known risk factors for AF, we found differences between the unmatched COVID-19 positive and negative cohorts regarding preexisting chronic renal failure, mitral valve disease, obesity, diabetes mellitus, hypertension, peripheral vascular disease, hyperlipidemia, smoking, and history of paroxysmal AF/AFl. Comparing the pre-pandemic with the COVID-19 positive cohort, differences were found for mitral valve disease, congestive heart failure, COPD, history of myocardial infarction, obesity, diabetes mellitus, hypertension, peripheral vascular disease, hyperlipidemia, smoking, and history of AF/AFl (see Table 1).

### Outcomes

In unmatched patients, AF occurred in 552 out of 4838 COVID-19 positive patients (11.4%), in 4718 out of 47,519 COVID-19 negative patients (9.9%) and in 2451 out of 26,368 pre-pandemic patients (9.3%). After matching, 192 out of 5005 pre-pandemic patients (3.8%) developed AF/AFl during the admission compared to 145 out of 2,283 COVID-19 positive patients (6.4%) leading to a crude odds ratio of 1.7 (95% CI 1.36, 2.12; *P* < 0.001) and a crude hazard ratio of 1.35 (95% CI 1.08, 1.68; *P* = 0.007; see Table 2). Comparing the matched COVID-19 negative and positive patients, 626 out of 11,004 (5.7%) of COVID-19 negative developed AF/AFl vs. 249 out of 3090 patients in the COVID-19 positive group (8.1%), resulting in a crude odds ratio of 1.45 (95% CI 1.25, 1.69; *P* < 0.001) and a crude hazard ratio of 1.24 (95% CI 1.07, 1.44; *P* = 0.0038).

**Table 2:** Matched patient outcomes.

	Pre-pandemic (n = 5005)	COVID-19 positive (n = 2283)	Odds ratio (95% CI)	*P*-value	COVID-19 negative (11,004)	COVID-19 positive (3,090)	Odds ratio (95% CI)	*P*-value
	Matched max. 3:1				Matched max. 6:1			
	Count (%)	Count (%)			Count (%)	Count (%)		
AF/AFl during admission	192 (3.8%)	145 (6.4%)	1.7 (1.36, 2.12)	**< 0.001**	626 (5.7%)	249 (8.1%)	1.45 (1.25, 1.69)	**< 0.001**
Death during admission	76 (1.5%)	163 (7.1%)	4.99 (3.78, 6.58)	**< 0.001**	228 (2.1%)	253 (8.2%)	4.22 (3.51, 5.07)	**< 0.001**

*AF* atrial fibrillation, *AFl* atrial flutter.

Significant values are in [bold].

Death during admission occurred in 544 out of 26,368 unmatched pre-pandemic patients (2.1%), 1139 out of 47,519 COVID-19 negative patients (2.4%) and 496 out of 4838 COVID-19 positive patients (10.3%). After matching, 76 out of 5005 pre-pandemic patients died during admission (1.5%) compared to 163 out of 2283 COVID-19 positive patients (7.1%) with a crude odds ratio of 4.99 (95% CI 3.78, 6.58; *P* < 0.001) and a crude hazard ratio of 2.08 (95% CI 1.51, 2.86; *P* < 0.001). 228 out of 11,004 COVID-19 negative patients (2.1%) died during admission after matching to 253 out of 3090 COVID-19 positive patients (8.2%) with a crude odds ratio of 4.22 (95% CI 3.51, 5.07; P < 0.001) and a crude hazard ratio of 2.23 (95% CI 1.82, 2.74; *P* < 0.001). The median (quartile 1 [Q1]–quartile 3 [Q3]) length of stay in the pre-pandemic patients was 3 days (2–6), in the COVID-negative patients 4 days (2–7), and in the COVID-positive patients 6 days (4–12). The difference in medians was 3 (95% CI 3, 3; *P* < 0.001) comparing unmatched COVID-19 positive and pre-pandemic patients, and 2 (95% CI 2, 2; *P* < 0.001) for COVID-19 positive vs. negative patients (“Supplementary Information”).

### Multivariable regression model

**Figure 2:**
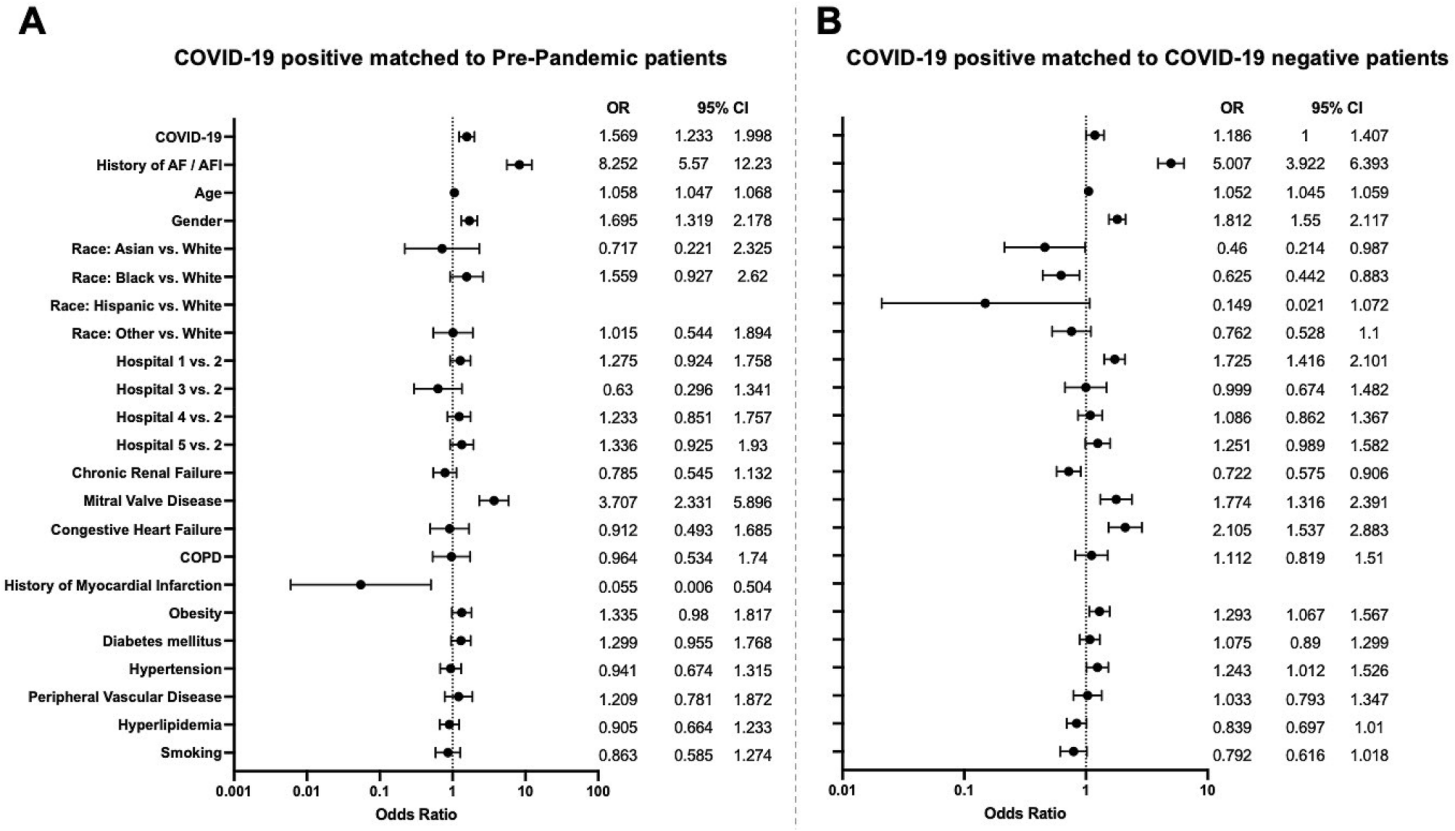
Multivariable logistic regression model and forest plots to determine the risk for atrial fibrillation after matching (blank rows due to insufficient data to analyze the variables Hispanic vs. White and History of myocardial infarction; “Race: Other vs White” includes Hispanic for the COVID-19 positive vs. Pre-Pandemic model; *AF* atrial fibrillation, *AFl* atrial flutter, *COPD* chronic obstructive pulmonary disease).

After adjusting for patient demographics and comorbidities, COVID-19 was associated with 1.19 (95% CI 1.00, 1.41) times the odds of developing AF (*P* = 0.0495; see Fig. 2) comparing matched COVID-19 positive and negative patients. Comparing COVID-19 positive with pre-pandemic patients resulted in an OR of 1.57 (95% CI 1.23, 2; *P* = 0.0003) in our sensitivity analysis.

History of paroxysmal AF or AFl was associated with an OR of 8.25 (95% CI 5.57, 12.23; P > 0.001) to develop AF/AFl during admission comparing COVID-19 positive and pre-pandemic patients, whereas the comparison of COVID-19 positive to matched COVID-19 negative patients led to an OR 5.01 (95% CI 3.92, 6.4; *P* < 0.001). Age was associated with 1.06 (95% CI 1.05, 1.07) and 1.05 (95% CI 1.05, 1.06) times the odds in the two matched groups of comparison for developing AF (both *P* = 0.001), while gender resulted in an OR of 1.7 (95% CI 1.32, 2.18) and 1.81 (95% CI 1.55, 2.12) respectively (both *P* = 0.001). Asian and black race led to a reduced odds for development of AF in the cohort of COVID-19 positive to negative patients with an OR of 0.46 (95% CI 0.21, 0.99; *P* = 0.0462) for Asian vs. White race and an OR of 0.63 (95% CI 0.44, 0.88; *P* = 0.00769) for Black vs. Whites. We furthermore found differences for the known risk factors chronic renal failure, mitral valve disease, congestive heart failure, history of myocardial infarction, obesity, and hypertension (see Fig. 2).

## Discussion

We were able to show an increased odds for COVID-19 positive patients to develop AF. This finding stresses the notion that COVID-19 is a cause for relevant extrapulmonary disease. Despite improvements in treatment, public measures of containment, and ongoing vaccination efforts, COVID-19 remains a challenge to public health. Until herd immunity is reached (either by vaccination or infection), it is likely going to remain a considerable problem worldwide. Despite almost two years of intensive research and media spotlight on COVID-19, the true extent of the sequelae of this disease is still unknown. COVID-19 may be capable of “many faces”^7^. In our study we aimed to further shed light into potential complications of myocardial involvement of COVID-19 and the development of AF, the most common arrhythmia which can lead to significant morbidity.

Severe pneumonia, acute respiratory distress syndrome and sepsis have previously been linked to new-onset AF^8,9^. Musikantow et al. were able to show that the rates of AF among hospitalized patients for COVID-19 was 10% and therefore not increased compared to patients with influenza^10^. In a Danish cohort the rates of AF have declined since imposing a national lockdown, but the rates of an adverse event (e.g. ischemic stroke) was higher during this time^11^. Case reports demonstrated that myocardial involvement can be apparent in critically ill patients suffering from COVID-19^1^. Specifically, serologic abnormalities were observed early during the pandemic among severely ill patients, showing an increase in troponin levels^12^. Myocardial biomarkers were shown to be of prognostic value for the disease process^13^. A study involving 416 hospitalized patients in Wuhan, China showed that cardiac injury was associated with a higher risk of in-hospital-mortality^14^. Cardiac MRI studies elucidated that myocardial involvement was present in 78% of patients, with ongoing inflammation in 60% patients despite recent recovery^15^. While virus-associated inflammation is not only capable of causing progression of pre-existing cardiac pathology, e.g. coronary artery disease, the clinical picture in COVID-19 mimics signs of de-novo myocarditis^16,17^. Myocardial inflammation itself can lead to complications like heart failure, dilated cardiomyopathy, and arrhythmias—with the most common form being AF.

Using data from our database involving more than 100,000 patients, we were able to show the direct association of COVID-19 and AF. Without adequate treatment, AF can lead to life-threatening complications, while rhythm-control, rate-control, and anticoagulation strategies are associated with adverse effects. In a significant portion of patients with AF, anticoagulation is needed to decrease the likelihood of thrombus formation, coming at the expense of an increased risk for bleeding. Thromboembolism is particularly relevant in COVID-19 as it is associated with hypercoagulability^18^: a substantial part of severe COVID-19 cases develop venous and arterial thromboembolic complications^19^. As the consequence of an acquired hypercoagulability, cryptogenic strokes can occur even among young COVID-19 patients^20^.

In our hospital-based database we used the PCR test for SARS-CoV-2 as a variable to identify patients of interest. The strength of our analysis is that we were able to collect the data of all inpatient encounters across the Mass General Brigham healthcare system from the beginning of the pandemic. Common risk factors for AF were adjusted for and helped to validate our results. As hospitalized patients during the height of the pandemics in 2020/21 (including COVID-negative patients) presented as a unique patient cohort, we included a sensitivity analysis to confirm our data: the results from pre-pandemic patients matched to COVID-19 positive patients confirmed our results and highlighted the association even further. In a cohort of 296 patients, Renda et al. showed that age was the most important risk factor for death from COVID-19, with AF being associated with a composite of cardiovascular complications^21^. Furthermore, Spinoni et al. showed in 637 patients that AF may be attributed to 30-day mortality in COVID-19 patients^22^.

Our study helps to direct the attention to adverse effects of COVID-19, warranting physicians to check for potential arrhythmogenic events. If, however, a dedicated screening for an irregular heartbeat (e.g. for patients tested positive for SARS-CoV-2 in an outpatient setting) can decrease the potential for complications needs to be determined. Also, lowering the threshold for anticoagulation in patients with high risk for AF needs to be investigated—pointing towards data of non-critically ill showing an increased probability of survival to hospital discharge by early therapeutic anticoagulation^23^. A thorough risk assessment for directed anticoagulation measures is furthermore prompted.

Several limitations have to be taken into consideration when interpreting our data. First, we had to rely on the accurate diagnosis of AF during the course of hospitalization. To account for improved detection of AF episodes, we used electrocardiogram, echocardiogram, telemetry documentation and other applicable types of cardiac reports. However, no uniform AF detection method was applied in these patients (like 24-h Holter monitor), therefore likely missing asymptomatic or short-lived episodes of AF. Previous literature suggests that accurate AF detection remains a major challenge^24^. Second, our hospitalized patients during the COVID-19 pandemic may have experienced a higher degree of disease severity or had other non-urgent medical causes leading to hospitalization. We therefore included a historic pre-pandemic cohort to verify our findings. Caution must be used when generalizing our findings to an outpatient cohort. Furthermore, we did not account for metrics of disease severity, nor did we control for different treatment regimes. Third, whether the onset of AF led to an increased incidence of complications cannot be answered from our data. Bigger, prospective studies with longer follow-up may help to substantiate the results, while further analyses of patient subgroups are needed to define patients at risk. Additional limitations apply due to the retrospective nature of our study, while we focused our analysis of confounders on known risk factors for the development of AF. It is furthermore unclear if AF due to COVID-19 remains a transient phenomenon or if it likely progresses into permanent AF. Similarly, it needs to be determined if it is strictly limited to a period of myocardial inflammation with subsequent termination. If an AF-directed treatment algorithm of patients with COVID-19 can help to overcome the burden of disease and therefore its high mortality needs to be studied further.

**Figure 3:**
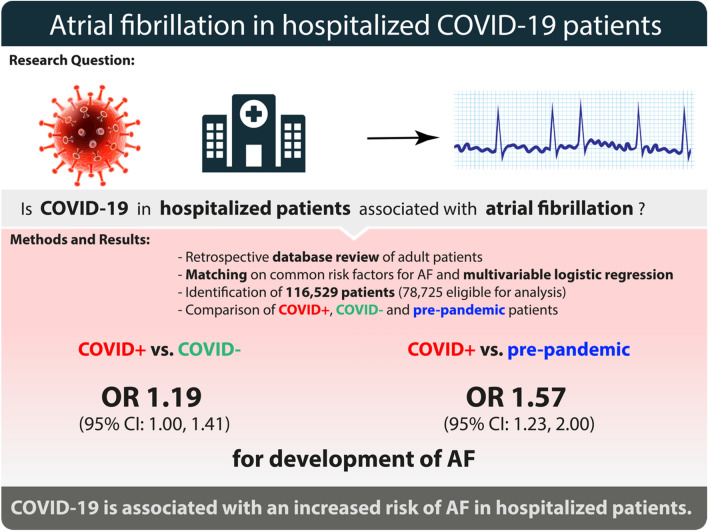
Summarizing figure of the study’s findings.

In conclusion, we were able to show an association of COVID-19 and the onset of AF in hospitalized patients. Our results (see Fig. 3) substantiate the need to further evaluate sequelae of COVID-19, while focusing on cardiovascular effects of the virus. Further studies are needed to elaborate on our findings, specifically to determine if dedicated screening and treatment strategies for COVID-19 associated AF may be beneficial.

## Methods

### Study design

The study was designed as a multi-center retrospective cohort analysis in five large academic tertiary care centers (Boston, USA). The analysis was planned and reported in accordance with the initiative for *Strengthening the Reporting of Observational Studies in Epidemiology* STROBE. using the suggested checklist for epidemiological cohort studies^25^. After approval by the local Institutional Review Board (Mass General Brigham IRB No. 2020P002679, August 20th, 2020), the hospital’s *Research Patient Data Registry* (RPDR) was queried. Informed consent was waived by the Institutional Review Board. It was not possible to involve patients or the public in the design, or conduct, or reporting, or dissemination plans of our research. The study was performed in accordance with ethics committee guidelines and the Declaration of Helsinki.

### Cohort definition

Our cohort included inpatients ≥ 18 years old from the affiliated Mass General Brigham institutions with hospital admission date between 12/1/2019 and 2/28/2021. Only the first hospitalization was included for patients with more than one inpatient admission during the study period. Due to a patient population in the COVID-negative group which was very different from hospitalized patients prior to the height of the COVID-19 pandemic, an additional retrospective cohort of pre-pandemic inpatients was included for analysis. Admissions were excluded if they spanned across the pre-pandemic and pandemic periods (i.e., admission date on or before 2/29/2020 and discharge date on or after 3/1/2020), the admission occurred during the pandemic era and no valid SARS-CoV-2 PCR test result was recorded in the electronic medical record (EMR) during admission, or patient sex or race was missing from the EMR.

### Study exposure

Our exposure of interest was COVID-19 diagnosed with a SARS-CoV-2 PCR test, with patients split into three groups: pre-pandemic, COVID-19 negative, and COVID-19 positive. Pre-pandemic was defined as patients with admission and discharge dates between 12/1/2019 and 2/29/2020. COVID-19 negative was defined as admission and discharge dates between 3/1/2020 and 2/28/2021, at least one valid SARS-CoV-2 PCR test result between admission and discharge, and no positive SARS-CoV-2 PCR test result with 30 days prior to admission through date of discharge. COVID-19 positive was defined as admission and discharge dates between 3/1/2020 and 2/28/2021, at least one valid SARS-CoV-2 PCR test result between admission and discharge, and at least one positive SARS-CoV-2 PCR test result with 30 days prior to admission through date of discharge.

### Primary outcome

Our primary outcome was atrial fibrillation (AF) or atrial flutter (AFl) during hospitalization based on electrocardiogram, echocardiogram, or other applicable types of cardiac reports.

### Secondary outcome

Secondary outcome analyses included mortality and length of hospitalization.

### Potential confounders

Measured potential confounders of the association between COVID-19 exposure group and AF or AFl during hospital admission included patient demographics (age at admission, sex, and race), patient comorbidities (renal failure, mitral valve disease, heart failure, chronic obstructive pulmonary disease [COPD], history of myocardial infarction, obesity, diabetes, hypertension, peripheral vascular disease, hyperlipidemia, current smoker, and history of stroke), and admitting hospital. Age at admission, sex, and race were identified using the EMR. History of AF or AFl were identified based on electronic electrocardiogram, echocardiogram, or other types of cardiac reports (e.g. cardiology consult note, telemetry, healthcare provider documentation), as well as International Classification of Diseases, Tenth Revision, Clinical Modification (ICD-10-CM) diagnosis codes. Obesity (within one year before admission) and current smoker (within 8 weeks before admission) were identified based on patient-reported social history in the EMR as well as ICD-10-CM diagnosis codes. The comorbidities renal failure, mitral valve disease, heart failure, COPD, history of myocardial infarction, diabetes, hypertension, peripheral vascular disease, hyperlipidemia, and history of stroke were identified using ICD-10-CM diagnosis codes.

### Patient and hospitalization characteristic comparisons

The magnitude and direction of differences in patient demographics, comorbidities, and hospital distribution between the COVID-19 positive vs. pre-pandemic and COVID-19 positive vs. COVID-19 negative groups were quantified as standardized differences^26^. Standardized differences with absolute value greater than or equal to 0.1 were taken to indicate a greater difference than would be expected by chance^27^. Demographics, comorbidities, and hospital distribution were also compared between the COVID-19 positive vs. pre-pandemic and COVID-19 positive vs. COVID-19 negative groups using two-sample *t*-tests for continuous variables and chi-square or Fisher’s exact tests for categorical variables. All comparisons were performed with the full cohort and the matched groups (matching described below).

### Outcome comparisons with full cohort

Median length of hospital stay was compared between COVID-19 exposure groups using quantile regression without covariate adjustment. Logistic regression without covariate adjustment was used to estimate the crude association of the COVID-19 exposure group with the odds of AF or AFl during hospitalization and with the odds of death during hospitalization. The adjusted association between the COVID-19 exposure group and the odds of AF or AFl during hospitalization was estimated using a multivariable logistic regression model with all measured potential confounders included as model covariates.

### Outcome comparisons with matched groups

Covariate matching was performed to establish overlap on demographics, comorbidities, and hospital distribution between the COVID-19 positive vs. pre-pandemic and COVID-19 positive vs. COVID-19 negative groups. First, COVID-19 negative patients were matched to COVID-19 positive patients in a maximum 6:1 ratio using a greedy algorithm. Matching criteria were age at admission ± 5 years and exact on all other potential confounders^28^. Second, the pool of COVID-19 positive patients who were successfully matched to at least one COVID-19 negative patient in the previous step were matched to pre-pandemic patients in a maximum 1:3 ratio using a greedy algorithm. All analyses performed in the full cohort were repeated for the matched groups. Since the COVID-19 positive patients matched to pre-pandemic patients were a subset of the COVID-19 positive patients matched to COVID-19 negative patients, separate models were run for comparison of each pair of groups. Comparisons of the matched groups did not account for matching because performing a matched analysis does not reduce bias in matched cohort studies^29^.

### Hypothesis testing and software

All statistical hypothesis tests were two-sided. All statistical analyses were performed using SAS software version 9.4 (SAS Institute, Cary, NC).

### Power analysis

A prior publication from one of the institutions included in this study reported that approximately 9% of hospitalized patients were COVID-19 positive between March and May 2020^30^. Prior literature has reported incidences of AF among hospitalized patients prior to the COVID-19 pandemic ranging from 9 to 10.7%^31–33^. Assuming an AF incidence of 9% in Covid-19 negative patients that make up 91% of the sample, an a priori* power* calculation found that a total of 4500 hospitalized patients would be required to detect a 50% increase in the incidence atrial fibrillation in COVID-19 positive vs. negative patients with 80% power at a two-sided alpha level of 0.05 with a chi-square test.

## Supplementary Information

Supplementary Information.

## Data Availability

The datasets generated during and/or analyzed during the current study are available from the corresponding author on reasonable request.
